# p38 Mediates Resistance to FGFR Inhibition in Non-Small Cell Lung Cancer

**DOI:** 10.3390/cells10123363

**Published:** 2021-11-30

**Authors:** Izabela Zarczynska, Monika Gorska-Arcisz, Alexander Jorge Cortez, Katarzyna Aleksandra Kujawa, Agata Małgorzata Wilk, Andrzej Cezary Skladanowski, Aleksandra Stanczak, Monika Skupinska, Maciej Wieczorek, Katarzyna Marta Lisowska, Rafal Sadej, Kamila Kitowska

**Affiliations:** 1 Department of Molecular Enzymology and Oncology, Intercollegiate Faculty of Biotechnology, University of Gdansk and Medical University of Gdansk, Debinki 1, 80-211 Gdansk, Poland; izabela.zarczynska@gumed.edu.pl (I.Z.); monika.gorska@gumed.edu.pl (M.G.-A.); acskla@gumed.edu.pl (A.C.S.); 2 Department of Biostatistics and Bioinformatics, Maria Sklodowska-Curie National Research Institute of Oncology, Gliwice Branch, Wybrzeze Armii Krajowej 15, 44-102 Gliwice, Poland; alexander.cortez@io.gliwice.pl (A.J.C.); Agata.Wilk@io.gliwice.pl (A.M.W.); 3 Center for Translational Research and Molecular Biology of Cancer, Maria Sklodowska-Curie National Research Institute of Oncology, Gliwice Branch, Wybrzeze Armii Krajowej 15, 44-102 Gliwice, Poland; Katarzyna.Kujawa@io.gliwice.pl (K.A.K.); Katarzyna.Lisowska@io.gliwice.pl (K.M.L.); 4 Department of Systems Biology and Engineering, Silesian University of Technology, 44-100 Gliwice, Poland; 5 Clinical Development Department, Celon Pharma S.A., Marymoncka 15, 05-152 Kazuń Nowy, Poland; aleksandra.stanczak@celonpharma.com (A.S.); maciej.wieczorek@celonpharma.com (M.W.); 6 Preclinical Development Departament, Celon Pharma S.A., Marymoncka 15, 05-152 Kazuń Nowy, Poland; monika.skupinska@celonpharma.com

**Keywords:** FGFR, p38, acquired resistance, lung cancer

## Abstract

FGFR signalling is one of the most prominent pathways involved in cell growth and development as well as cancer progression. FGFR1 amplification occurs in approximately 20% of all squamous cell lung carcinomas (SCC), a predominant subtype of non-small cell lung carcinoma (NSCLC), indicating FGFR as a potential target for the new anti-cancer treatment. However, acquired resistance to this type of therapies remains a serious clinical challenge. Here, we investigated the NSCLC cell lines response and potential mechanism of acquired resistance to novel selective FGFR inhibitor CPL304110. We found that despite significant genomic differences between CPL304110-sensitive cell lines, their resistant variants were characterised by upregulated p38 expression/phosphorylation, as well as enhanced expression of genes involved in MAPK signalling. We revealed that p38 inhibition restored sensitivity to CPL304110 in these cells. Moreover, the overexpression of this kinase in parental cells led to impaired response to FGFR inhibition, thus confirming that p38 MAPK is a driver of resistance to a novel FGFR inhibitor. Taken together, our results provide an insight into the potential direction for NSCLC targeted therapy.

## 1. Introduction

Lung cancer is the most common cause of cancer-related deaths in men and women, with 2,206,771 new cases and 1,796,144 deaths in 2020 worldwide [1]. Non-small cell lung carcinoma (NSCLC), which comprises approximately 84% of all lung cancers, is categorised into adenocarcinoma (ADC, approximately 40–50% of all cases), squamous cell carcinoma (SCC, approximately 20–30% of all cases), and large cell carcinoma (LCC, 10% of all cases). Gene alterations and rearrangements in *EGFR*, *ALK*, *ROS1*, *BRAF*, or *NTRK* typical for ADC were discovered in the last two decades, which led to the development of targeted therapies using tyrosine kinase inhibitors (TKI) [2]. Such drugs, applied as an alternative to conventional chemotherapy and radiotherapy in first-line treatment of advanced ADC, have significantly improved clinical outcomes. Contrary to ADC, SCCs harbour distinct types of gene alterations: amplifications (*MET*, *HER2*, and *FGFR*) [3] and gene mutations (*CDKN2A*, *PTEN*, *KEAP1*, *MLL2*, *HLA*-*A*, *NFE2L2*, *NOTCH1*, and *RB1*) [4]. Therefore, several clinical trials are underway, developing the targeted therapies specifically for lung squamous cell carcinomas (SCC).

The FGFR family consists of four transmembrane receptor tyrosine kinases (FGFR1-4) activating multiple signalling pathways, including RAS/RAF/MAPK, PI3K/AKT, and STAT involved in the regulation of proliferation, cell survival, migration, and invasion [5]. FGFR-related genomic alterations, i.e., gene amplification, chromosomal translocation, gain-of-function mutations, and gene fusions, lead to constitutive receptor activation or enhanced signalling [6,7]. FGFR1 amplification is one of the most common genomic alterations in SCC, occurring in 10–20% of cases. Several studies demonstrated that SCCs also harbour FGFR2 and FGFR3 fusions [7,8]. Since deregulated FGFR signalling has been implicated in oncogenesis and cancer progression, FGFR emerged as a promising target for anti-cancer therapies [9]. A few FGFR tyrosine kinase inhibitors (TKIs) have been approved for clinical use, and several are currently undergoing preclinical and clinical investigation in various FGFR-associated tumours (NCT02965378, NCT03762122, NCT02154490, NCT03827850). Although small-molecule inhibitors of FGFR activity represents a promising anti-cancer strategy, emerging resistance to applied drug remains a growing challenge.

So far, several mechanisms of acquired resistance to therapies targeting FGFR have been presented. In general, acquired resistance to TKIs can develop either as a result of secondary mutations within the ATP-binding domain, preventing the receptor from inhibitor binding, or through activation of alternative signalling pathways that circumvent the FGFR signalling cascade [10,11,12,13]. In vitro studies performed in lung cancer cell lines revealed that MET upregulation followed by reactivation of the ERK/MAPK pathway is involved in the development of resistance to BGJ398 [14]. Moreover, the AKT pathway was shown to mediate resistance to BGJ398 in lung and urothelial cancer cell lines [15]. These results are in line with studies indicating that incomplete suppression of the key survival pathways: PI3K/AKT and MAPK by BGJ398 or PD173074 in lung and colorectal cancer cell lines may be associated with acquired resistance to FGFR inhibitors [16].

In this study, we analysed the sensitivity of a panel of lung cancer cell lines to CPL304110 (Celon Pharma, Poland), a novel pan-FGFR inhibitor that is currently in phase I of a clinical trial in adults with advanced solid malignancies (NCT04149691) [17]. We identified NCI-H1581 and NCI-H1703 non-small cell lung cancer cell lines as highly sensitive to inhibition of FGFR. We found that despite genomic and transcriptomic divergence between parental cells, CPL304110-resistant variants of these cell lines displayed increased expression of the genes encoding members of the p38 signalling pathway. Moreover, the overexpression of p38 kinase resulted in resistance to CPL304110 in parental cells, while inhibition of p38 MAPK resensitised resistant cells to CPL304110 treatment. These confirmed the importance of p38 MAPK in the process of acquisition of resistance to inhibition of FGFR signalling in NSCLC cell lines.

## 2. Materials and Methods

### 2.1. Cell Lines and Cell Culture Reagents

DMS114, NCI-H1581, NCI-H1703, NCI-H2170, and NCI-H520 cell lines were obtained from ATCC. NCI-H1581 cells were routinely maintained in DMEM/F12; DMS114 in Waymouth’s MB752/1; whereas NCI-H1703, NCI-H2170, and NCI-H520 were maintained in RPMI 1640 medium. All culture media contained 10% of FBS and penicillin/streptomycin (100 U/mL/100 μg/mL). Cells were grown at 37 °C in a humidified atmosphere of 5% CO_2_. All culture media and corresponding supplements were purchased from Merck KGaA (Darmstadt, Germany) or Biowest (Riverside, MO, USA). CPL304110 (WO/2014/141015) inhibitor was provided by Celon Pharma S.A., Poland [17]. SB202190 and SB203580 inhibitors were purchased from Selleckchem (Houston, TX, USA).

### 2.2. Cell Proliferation Assay

Cell viability was estimated using the 3-(4,5-dimethylthiazol-2-yl)-2,5-diphenyltetrazolium bromide (MTT) colorimetric assay Merck KgaA (Darmstadt, Germany). Cells were seeded in 96-well plates in triplicates and on the following day treated with DMSO or indicated inhibitor for 48 and 96 h. MTT stock solution was added to each well so that the final concentration of MTT in the medium was 0.5 mg/mL. After 2 h incubation at 37 °C, the medium was discarded, and MTT formazan was dissolved in DMSO. The absorbance was measured at 590 nm using a microplate reader.

### 2.3. Clonogenic Assay

Cells were seeded in 6-well plates. On the following day, the medium was replaced with a regular medium or medium containing indicated inhibitors. Media were changed every three days. Cells were cultured for 10–14 days, depending on the cell line, fixed with 4% paraformaldehyde and stained with 0.4% crystal violet.

### 2.4. Culturing Cells in Three-Dimensional BD Matrigel^®^

Cell growth in three-dimensional (3D) BD Matrigel^®^ (BD Matrigel Matrix Growth Factor Reduced, BD Bioscience, Corning, NY, USA) was carried out as previously described [18]. Cells were cultured in regular medium or medium containing indicated inhibitors. Media were replaced every three days. After 14 days of culture, cell growth was evaluated by measuring colonies size (at least 100) using ZEISS PrimoVert microscope and ImageJ software.

### 2.5. Generation of CPL304110-Resistant Cell Lines

To develop resistance to the FGFR inhibitor, CPL304110, NCI-H1581, and NCI-H1703 cell lines were exposed to increasing concentrations of CPL304110 (starting from 50 nM). Cells were maintained in a medium containing the inhibitor, which was replaced every three days. When the growth kinetics of treated cells were similar to wild-type cells, the concentration of CPL304110 was increased until a final concentration of 2.5 μM for NCI-H1581 and 5 μM for NCI-H1703 was achieved. After 4-6 months of such culture, resistant cells were established and termed NCI-H1581R and NCI-H1703R.

### 2.6. Overexpression of p38

To generate cells overexpressing p38 MAPK NCI-H1581 and NCI-H1703 cells were seeded onto 6 cm plates and, after 24 h, transfected with pMT3-p38-HA plasmid (Addgene, #12658) using Lipofectamine 3000 (Invitrogen, Thermo Fisher Scientific, MA, USA). The overexpression was confirmed with Western blotting.

### 2.7. Western Blotting Analysis

For Western blotting analysis, cells were harvested at 60–70% confluency and lysed with 2x concentrated Laemmli buffer supplemented with 2 mM PMSF, 10 μg/mL aprotinin, 10 μg/mL leupeptin, 5 mM EGTA, 1 mM EDTA, 2 mM Na_4_P_2_O_7_, 5 mM NaF, and 5 mM Na_3_VO_4_. Samples with equal amounts of protein were loaded per well, resolved in SDS-PAGE, and then transferred onto a nitrocellulose membrane. The membranes were blocked for 1 h in 5% skimmed milk and probed with specific primary antibodies at 4 °C. Antibodies for anti-FGFR1 (sc-57132), anti-FGFR3 (sc-13121), anti-FGFR4 (sc-136988), and anti-FRS2-α (sc-17841) were obtained from Santa Cruz Biotechnology (Dallas, TX, USA). The antibody against β-actin (A5316) was obtained from Merck KGaA (Darmstadt, Germany). All the remaining antibodies were from Cell Signaling Technology (Danvers, MA, USA): anti-Akt-Ser473 (#9271) anti-Akt (#92720), anti-CDK4 (#12790), anti-CDK6 (#3136), anti-Erk1/2-Thr202/Tyr204 (#4377), anti-Erk1/2 (#9102), anti-FGFR-Tyr653/654 (#3471), anti-FGFR2 (#23328), anti-FRS2-α-Tyr196 (#3864), anti-FRS2-α-Tyr436 (#3861), anti-PLC-γ-1-Tyr783 (#2821), anti-PLC-γ-1 (#2822), anti-p27-Kip1 (#3686), anti-p38-MAPK-Thr180/Tyr182 (#9211), anti-p38-MAPK (#9212), anti-Rb-Ser780 (#9307). Appropriate secondary Alexa Fluor^®^-conjugated antibodies (680 or 790 nm) (Jackson ImmunoResearch, #111-625-144, #715-655-150) and Odyssey^®^ CLx imaging system (LI-COR^®^ Biosciences, NE, USA) were used to detect protein bands.

### 2.8. Cell Cycle Analysis

The cell cycle was analysed by quantification of DNA content using flow cytometry. Cells were fixed in 70% ethanol for 24 h at −20 °C, RNase A (1 mg/mL) was added, EURX Ltd. (Gdansk, Poland), and cells were stained with propidium iodide (2,5 µg/mL) (PI; #P4170, Sigma-Aldrich; Merck KGaA). The cell cycle was analysed with FACSCalibur™; Becton Dickinson and Company (San Jose, CA, USA). The results were analysed using the CellQuest™ Pro Software version 6.0 Becton Dickinson and Company (San Jose, CA, USA).

### 2.9. Array-Based Comparative Genomic Hybridisation (aCGH)

The aCGH was used to reveal copy number changes (amplifications and deletions) in the genome of sensitive versus resistant cells (NCI-H1581 vs. NCI-H1581R and NCI-H1703 vs. NCI-H1703R). DNA from the cells was isolated using GeneJET Genomic DNA Purification Kit, Thermo Fisher Scientific (Waltham, MA, USA). Genomic DNA was analysed by hybridisation to the 60K SurePrint G3 Unrestricted CGH arrays, Agilent Technologies, Inc. (Santa Clara, CA, USA) service provided by Genomed S.A., Warsaw, Poland. Normal human Caucasian male genome GRCh38 (hg38) was used as the reference.

### 2.10. aCGH Data Analysis

Raw image files were preprocessed using Agilent Feature Extraction software (version 11.0.1.1). Data were checked for quality, and features were extracted for CGH_1100_Jul11 protocol. Further analysis was conducted with Bioconductor package rCGH version 1.20.0 released on 28 October 2020 according to standard protocol dedicated for Agilent dual-colour hybridisation chips [19]. First, the signals were adjusted for GC content and cy3/cy5 bias, after which the log2 relative ratios (LRR) could be computed. Next, the genome profiles were segmented using the Circular Binary Segmentation algorithm, and finally, the LRRs were centred using an expectation–maximisation algorithm. Results were visualised using Rcircos version 1.2.1 released on 12 March 2019 and pheatmap version 1.0.12 released on 4 January 2019 (https://cran.r-project.org/web/packages/pheatmap/index.html, accessed on 28 October 2021) R packages [20]. Genes were considered differential between variants if the absolute value of LRR difference was greater than 0.5. All analyses were performed using R environment for statistical computing version 4.0.3 “Bunny-Wunnies Freak Out” released on 10 October 2020 (R Foundation for Statistical Computing, Vienna, Austria, http://www.r-project.org, accessed on 28 October 2021).

### 2.11. Transcriptome Sequencing (RNA-Seq)

For RNA-seq experiment, NCI-H1581 and NCI-H1703 cells (sensitive and resistant cell line variants) were seeded onto 6 cm-diameter dishes. The next day, the medium was replaced with a fresh medium, and after 24 h, cells were harvested. This procedure was repeated to obtain a second replicate. Total RNA was extracted from the cells using RNeasy Plus Mini Kit, Qiagen (Hilden, Germany) with simultaneous DNase I digestion, according to the manufacturer’s instructions. RNA purity and concentration were estimated with a Nanodrop ND-2000 spectrophotometer, Thermo Fisher Scientific (Waltham, MA, USA). RNA quality was assessed using the 2100 Bioanalyzer with the RNA 6000 Nano Kit, Agilent Technologies (Santa Clara, CA, USA). All the samples had an RNA integrity number (RIN) above 7.0. cDNA library preparation and transcriptome sequencing were completed by Genomed, Warsaw, Poland. RNA-seq was performed using the Illumina HiSeq4000 Platform with the standard paired-end protocol (58 mln paired reads, 100 bp read length).

### 2.12. RNA-seq Data Analysis

After standard quality control, the raw sequencing data were quantified using the Salmon tool against the reference genome GRCh38 (hg38) [21]. Quantified transcripts were imported into the R environment with tximport [22]. Low-abundance genes were pre-filtered, keeping only rows with at least 10 reads total. Gene counts were normalised using the median-of-ratios method [23]. For unsupervised analysis, regularised logarithm (rlog) transformed data were used. Principal Component Analysis (PCA) was applied to assess the main sources of variability in data. Hierarchical clustering of 500 genes exhibiting the largest overall variance was performed to explore relationships between samples–heatmaps were generated with pheatmap R package (https://cran.r-project.org/web/packages/pheatmap/index.html, accessed on 28 October 2021). Both the unsupervised methods revealed significant differences between cell lines, effectively overshadowing differences between variants, for which reason further analysis was performed separately for the two cell lines. Differentially expressed genes were identified using DESeq2 package [23] version 1.30.1 released on 20 February 2021, with FDR adjusted (Benjamini–Hochberg correction) *p*-value cut-off 0.1. Overrepresented signalling pathways were identified with Gene Set Enrichment Analysis (GSEA) method [24] implemented in Bioconductor package clusterProfiler version 3.14.3 released on 8 January 2020 [25]. Gene sets between 10 and 700 bp were considered, and the permutation number was set to 10,000. Pathways were downloaded from MsigDB version 7.3 [26]. For global analysis, the C2:CP (Curated gene sets: Canonical pathways) collection was used. All analyses were performed using R environment for statistical computing version 4.0.3 “Bunny-Wunnies Freak Out” released on 10 October 2020 (R Foundation for Statistical Computing, Vienna, Austria, http://www.r-project.org, accessed on 28 October 2021).

### 2.13. Statistical Analysis

All data are expressed as mean ± SD from at least three independent experiments. Comparative data were analysed with the unpaired Student’s *t*-test using the Statistica™ software, v.10; TIBCO Software Inc. (Palo Alto, CA, USA). Two-sided *p* ≤ 0.05 was considered a statistically significant difference.

## 3. Results

### 3.1. Differential Response of NSCLC Cell Lines to CPL304110 Inhibitor

**Figure 1:**
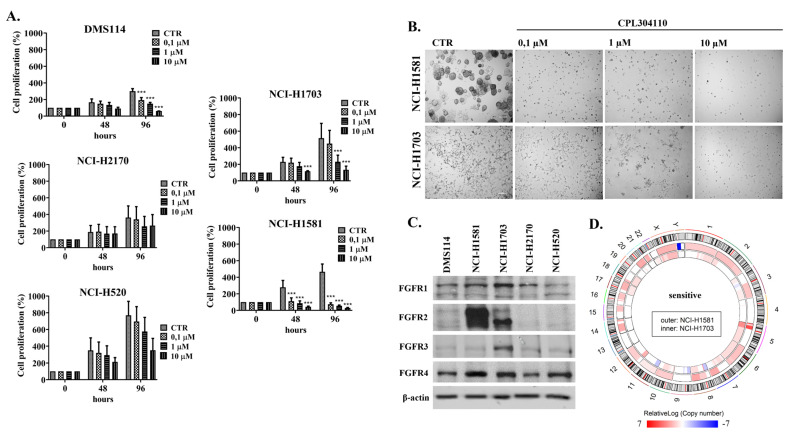
Lung cancer cell line response to a novel FGFR inhibitor. (**A**) DMS114, NCI-H1581, NCI-H1703, NCI-H2170, and NCI-H520 cell lines were exposed to the indicated CPL304110 concentrations for 48 and 96 h. The anti-proliferative effect of the inhibitor was assessed using the MTT cell viability test. Data are expressed as mean ± SD, *** *p* ≤ 0.001 compared to non-treated cells, n = 3. (**B**) NCI-H1581 and NCI-H1703 cells were grown in 3D BD Matrigel^®^ for 14 days in the presence of CPL304110 in indicated concentrations. Representative pictures were taken. Scale bar represents 100 µm, n = 3. (**C**) Western blot analysis of FGFR1-4 protein expression was performed for lysates of all five lung cancer cell lines. Experiments were conducted in triplicates. Representative blots are shown. (**D**) The circos plot depicts aCGH-derived DNA copy number profiles (relativeLog) of the analysed lung cancer cell lines. The outer circle represents chromosome cytobands (centromeres are shown as red bars) of the reference human genome GRCh38; middle and inner circle show a copy number changes in the NCI-H1581 and NCI-H1703 cells (respectively), in comparison to the reference genome. Numbers and letters on the outside indicate chromosomes.

Approximately 20% of all non-small cell lung cancer cases are characterised by FGFR1 amplification, indicating FGFR as a promising target for anti-cancer therapies [16]. Therefore, we analysed the response of five lung cancer cell lines to the new FGFR inhibitor, CPL304110. Evaluation of cell proliferation revealed that NCI-H1581 and NCI-H1703 cells strongly responded to CPL304110, showing a significant growth reduction with IC50 < 1 µM, while DMS114, NCI-H2170, and NCI-H520 cells were less sensitive to FGFR inhibition (IC50 ≥ 1 µM) (Figure 1A). In accordance with these results, significant CPL304110-mediated 3D growth inhibition, even in 0.1 µM, relatively low drug concentration, was found for both cell lines (Figure 1B). Since NCI-H1703 cells exhibit highly invasive growth and do not form classical spheroids in 3D culture, the quantitative growth analysis was possible only for the NCI-H1581 cell line (Figure S1). Further investigation revealed that response to CPL304110 correlated with FGFR protein expression, as Western blotting analysis showed the highest FGFR1-4 expression levels in NCI-H1581 and NCI-H1703 among analysed cell lines (Figure 1C).

Although NCI-H1581 and NCI-H1703 cells were both found sensitive to CPL304110, array based-comparative genome hybridisation (aCGH) analysis revealed significant genetic differences between them. Both cell lines showed significant copy number variation (CNV) compared to the normal human genome, which was distinct for each cell line, as illustrated by the circos plot (Figure 1D). Although the NCI-H1581 genome was characterised by the prevalence of gain of DNA copy number events, loss of copy number was observed only within chromosome Y. On the other hand, the NCI-H1703 genome was rich in both gains and losses of DNA copy number.

### 3.2. CPL304110 Induces Cell Cycle Arrest in Sensitive Cells

**Figure 2:**
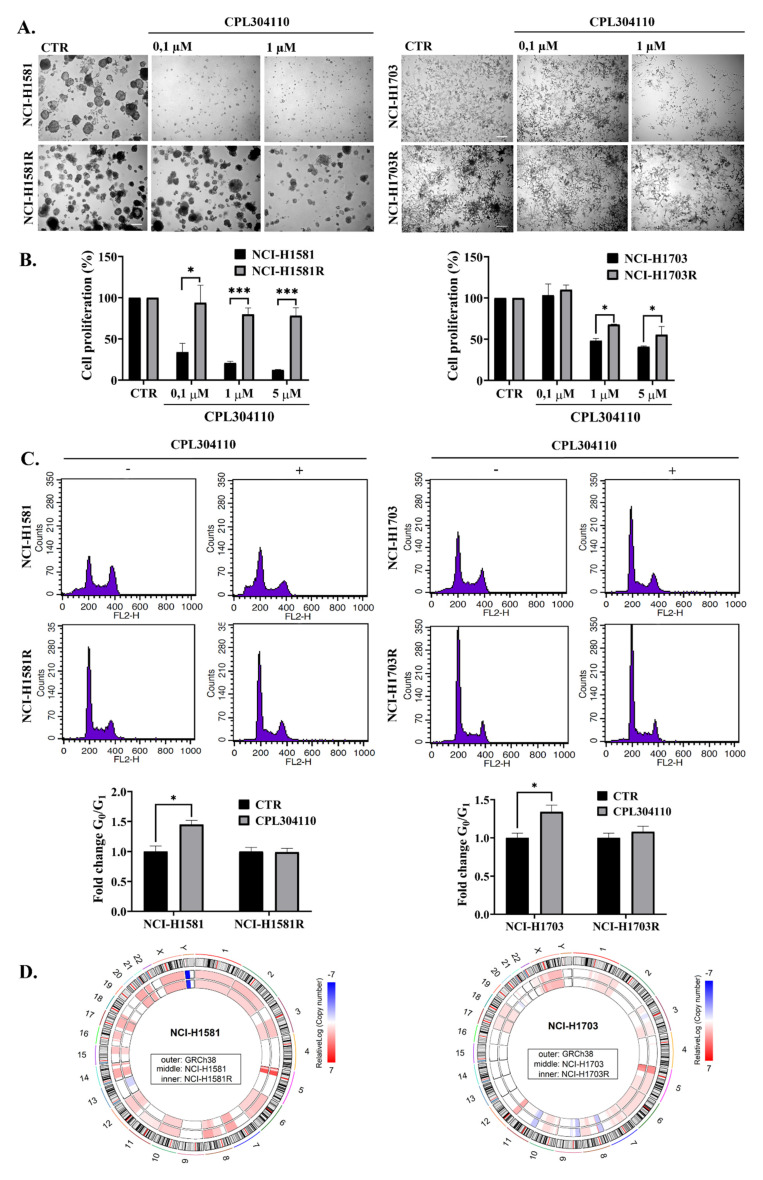
CPL304110-mediated cell cycle arrest. Resistance to CPL304110 was induced in NCI-H1581 and NCI-H1703 by chronic exposure to CPL304110. (**A**) Response of sensitive and resistant cells to the FGFR inhibitor was analysed in 3D BD Matrigel^®^. After 14 days of culture, representative pictures were taken. Scale bar represents 100 µm, n = 3. (**B**) Proliferation of sensitive and resistant cells in the presence of CPL304110 was evaluated with MTT test. Data are expressed as mean ± SD, * *p* ≤ 0.01, *** *p* ≤ 0.001, n = 3. (**C**) Cell cycle analysis of CPL304110-treated cells. Sensitive and resistant cell variants were serum-starved and subsequently treated with CPL304110 (0.1 µΜ for NCI-H1581 and NCI-H1581R; 1 µΜ for NCI-H1703 and NCI-H1703R) for 48 h. Bar graphs represent the fold change of cells arrested in the G0/G1 phase. Data are expressed as mean ± SD, * *p* ≤ 0.01, n = 3. (**D**) Circos plots representing a copy number variation (relativeLog) in NCI-H1581 vs. NCI-H1581R (left circos) and NCI-H1703 vs. NCI-H1703R (right circos). The outer circle represents chromosome cytobands (the centromeres are shown as a red bar) of the reference human genomeGRCh38 middle and the inner circle represents a copy number variation in the sensitive and resistant variant (respectively), in comparison to reference genome. Numbers and letters on the outside indicate chromosomes.

CPL304110-resistant cell line variants (NCI-H1581R and NCI-H1703R) were developed to investigate the mechanism of acquired resistance to FGFR inhibition. Resistance to CPL304110 has been confirmed for both cell lines with analysis of 3D cell growth in BD Matrigel^®^ (Figure 2A and Figure S2A) and proliferation assay (Figure 2B). Analysis of cell cycle revealed that it was affected by inhibition of FGFR only in sensitive cells.

CPL304110-induced cell cycle arrest in G0/G1 phase was observed for NCI-H1581 and NCI-H1703 but not in resistant cell variants (Figure 2C). This has been supported by analysis of CPL304110 impact on expression/activity of proteins involved directly in regulation of cell cycle. Upon CPL304110 treatment, sensitive cells displayed a downregulated expression of CDK4 and CDK6 together with a decrease in Rb phosphorylation and upregulation of p27, which is a well-known cell cycle inhibitor. For both resistant variants, treatment with CPL304110 did not affect the level of any of the cell cycle-related proteins (Figure S2B).

In Principal Component Analysis, the main source of variance in gene expression profile was related to the type of cell line. First Principal Component (PC1) was responsible for 74% of the variance in gene expression and was related to the NCI-H1581 vs. NCI-H1703 difference. PC2 was identified as related to NCI-H1581R vs. NCI-H1581 difference (13% of variance), while PC3 was related to NCI-H1703R vs. NCI-H1703 difference (only 5% of variance) (Figure S2C). Additionally, hierarchical clustering showed that samples group preferentially by cell line type but not by their sensitivity to FGFR inhibition (resistant vs. sensitive) (Figure S2D). The comparison of CNV patterns between sensitive and resistant variants revealed several differences, which might be related to the acquisition of resistance to CPL304110 (Figure 2D). Interestingly, different changes were observed in each cell line. The majority of genetic events observed in NCI-H1581R were connected with a reduction of DNA copy number compared to NCI-H1581 but still resulting in more DNA copies than in normal reference genome. This trend was observed in large parts of chromosomes 2, 3, 6, 8, 12, and 15 and small portions of chromosomes 10 and 18. On the contrary, NCI-H1703R showed an increase in DNA copy number within the portion of chromosomes 3, 5, 8, 22, and X, normalisation of DNA copy number in the fragment of chromosomes 3, 4, and 5, and decrease of DNA copy number in part of chromosome 11. Such a diverse pattern of copy number alterations accompanying acquired resistance to CPL304110 may suggest different mechanisms of drug resistance in analysed cell lines.

### 3.3. Identification of Potential Biomarker of Acquired Resistance to CPL304110 Inhibitor

**Figure 3:**
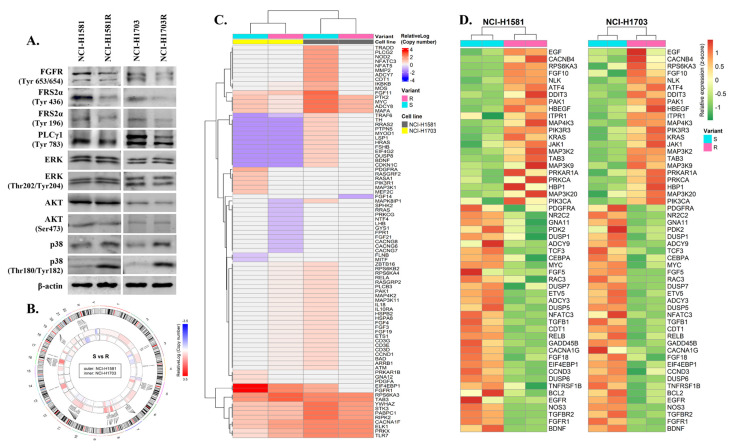
Involvement of p38 kinase in resistance to FGFR inhibition. (**A**) Protein expression/phosphorylation levels of FGFR and its direct downstream effectors were analysed with Western blot. Experiments were conducted in triplicates. Representative blots are shown. (**B**) A circos plot showing a copy number variation (relativeLog) in resistant variant of NCI-H1581 (outer circle) and NCI-H1703 (inner circle) cell lines in comparison to respective sensitive variant. Enlarged circos plot with a legible gene names is shown in Supplementary Materials. (**C**) A heat map showing genes with copy number variation (CNV) between sensitive versus resistant variant (data subjected to rlog transformation). (**D**) A heat map showing differentially expressed genes between sensitive versus resistant cell line variants (only genes with the mutual direction of change in both analysed cell lines are shown; data subjected to rlog transformation).

In further experiments, the molecular mechanism of acquired cell resistance to CPL304110 was investigated. Multiple studies demonstrated that the mechanisms of acquired resistance to TKIs are associated with reactivation of downstream signalling, which initially is switched off by applied inhibitors [27,28]. When CPL304110-sensitive cells were compared to their resistant variants, it was found that the phosphorylation of FGFR and its direct downstream effector proteins Fibroblast Growth Factor Receptor Substrate 2-α (FRS2-α) and phospholipase C-γ-1 (PLC-γ-1) were decreased in NCI-H1581R and NCI-H1703R (Figure 3A). Additionally, we investigated the expression and phosphorylation levels of AKT, ERK, and p38 MAPK, which have been previously implicated in mediating the acquired resistance to RTK inhibitors in lung cancer cells [14,15,29,30]. For both CPL304110-resistant cell lines, no changes in expression level, as well as phosphorylation, were observed for AKT and ERK compared to corresponding sensitive variants.

Interestingly, NCI-H1581R and NCI-H1703R demonstrated an increased expression and phosphorylation of p38 kinase, indicating its upregulation as a possible common mechanism of acquired resistance to FGFR inhibition. Additionally, Western blot analysis of p38 expression level in all five originally investigated lung cancer cell lines (Figure S3) confirmed that it is related to acquired resistance and does not correlate with the initial cells response to CPL304110.

To investigate the genomic changes related to CPL304110 resistance, a copy number analysis of p38-related genes was performed (Figure 3B,C). In both NCI-H1581R and NCI-H1703R, the analysis revealed a decrease in copy number of the following genes: *MYC*, *PTK2*, *ADCY8*, *MAFA*, *MAPK8IP1*, *EIF4EBP1*, and *FGFR1*. Our RNA-sequencing analyses of p38 signalling (Figure S4A–F) revealed a panel of 54 genes common for both analysed cell lines with the same pattern of changes in expression following the acquisition of resistance to CPL304110. Among these genes, members of MAPK, TGFβ, PI3K, EGF, and FGF pathways were found, as well as genes involved in the regulation of cell cycle and apoptosis (Figure 3D). Interestingly, three genes (*MYC*, *EIF4EBP1*, and *FGFR1*) showed a decrease in both copy number and expression in resistant cell variants.

### 3.4. p38 Mediates Resistance to FGFR Inhibition in Lung Cancer Cells

**Figure 4:**
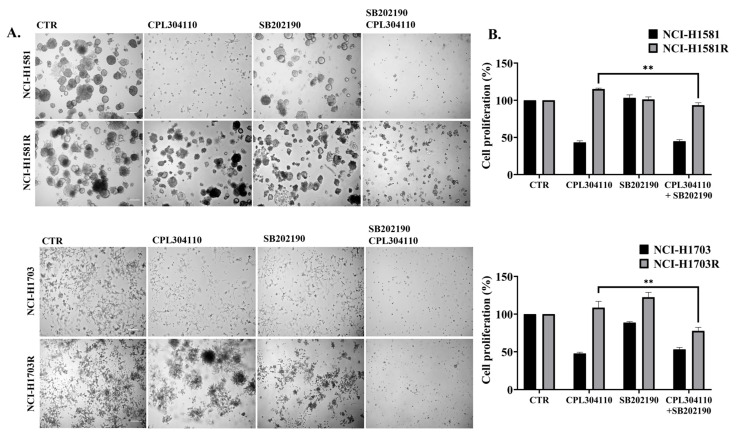
p38 activity mediates in CPL304110-induced cell growth inhibition. (**A**) Sensitive and resistant variants of NCI-H1581 and NCI-H1703 cells were grown with CPL304110 (0.1 µΜ for NCI-H1581 and NCI-H1581R; 1 µΜ for NCI-H1703 and NCI-H1703R) and/or SB202190 (2 µM) in 3D BD Matrigel^®^. Cell growth was measured with ImageJ software after 14 days of culture. Representative pictures were taken. Scale bar represents 100 µm, n = 3. (**B**) Proliferation analysis was evaluated by MTT in sensitive and resistant cells exposed to CPL304110 (0.1 µΜ for NCI-H1581 and NCI-H1581R; 1 µΜ for NCI-H1703 and NCI-H1703R) and/or SB202190 (2 µM) for 96 h. Data are expressed as mean ± SD, ** *p* ≤ 0.005, n = 3.

Previous results led to an assumption that p38 kinase may be involved in the mechanism of resistance to CPL304110. To further verify whether p38 mediates resistance to FGFR inhibitor, we analysed how SB202190, a chemical inhibitor of p38, affects cell growth in 3D BD Matrigel^®^, proliferation, and response to CPL304110. The inhibitory effect of SB202190 on p38 activity was analysed with Western blot (Figure S5). Interestingly, we observed that the inhibition of p38 resulted in reduced cell growth in 3D and impaired the colony formation of CPL304110-resistant cells. Furthermore, the inhibition of p38 with SB202190 restored the sensitivity of NCI-H1581R and NCI-H1703R cells to CPL304110 as dual inhibition of FGFR and p38 led to reduced cell growth in 3D and proliferation (Figure 4A,B and Figure S6), as well as impaired colony formation (Figure S7). In concordance with these results, we observed reduced cell growth in 3D upon treatment with another commercially available p38 inhibitor, SB203580 (Figure S8). These results further indicated p38 involvement in CPL304110 resistance.

**Figure 5:**
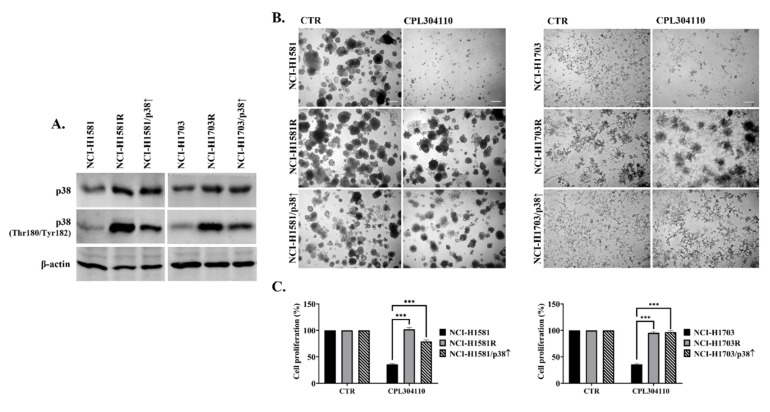
p38 MAPK overexpression induces resistance to FGFR inhibition. (**A**) p38 kinase overexpression was established in NCI-H1581 and NCI-H1703 cells and confirmed with Western blot. Experiments were conducted in triplicates. Representative blots are shown. (**B**) Cell growth in 3D BD Matrigel^®^ and (**C**) cell proliferation in the presence of CPL304110 (0.1 µΜ for NCI-H1581, NCI-H1581R, NCI-H1581/p38↑; 1 µΜ for NCI-H1703, NCI-H1703R, NCI-H1703/p38↑) was assessed. Cells were cultured in 3D BD Matrigel^®^ for 14 days. Representative pictures were taken. Scale bar represents 100 µm, n = 3. Cell proliferation was assessed using MTT viability assay after 96 h. Data are expressed as mean ± SD, *** *p* ≤ 0.001, n = 3.

As inhibition of p38 resensitised CPL304110-resistant cells to FGFR inhibition, we investigated whether the overexpression of p38 was sufficient to confer resistance to CPL304110 in sensitive cells. We established two cell lines (NCI-H1581/p38↑ and NCI-H1703/p38↑) with a stable overexpression of p38 (and concomitant increase in p38 phosphorylation) (Figure 5A) to mimic p38 status in CPL304110-resistant cells. Interestingly, the ectopic overexpression of p38 led to significantly impaired sensitivity to CPL304110, as the proliferation and 3D growth of NCI-H1581/p38↑ and NCI-H1703/p38↑ were not or barely affected by FGFR inhibition (Figure 5B,C and Figure S9). The obtained results indicate that acquired resistance to FGFR inhibition in lung cancer cells is mediated by p38, suggesting that dual inhibition of FGFR and p38 might serve as a feasible direction in lung cancer targeted therapy.

## 4. Discussion

Deregulation of FGFR signalling has a significant impact on cancer development and progression. Therefore, extensive preclinical and clinical studies were undertaken to introduce the new generation of selective FGFR inhibitors to anti-cancer therapy. FGFR1 amplification is one of the most common genomic alterations in SCC. Therefore, FGFR1 has been considered as one of the promising therapeutic targets [31,32,33,34]. Herein, we present preclinical studies for a novel FGFR inhibitor, CPL304110, which is currently in phase I of clinical trials in adults with advanced solid malignancies. After the analysis of response to CPL304110 in the panel of five lung cancer cell lines, the two most sensitive, NCI-H1581 and NCI-H1703, were used for further studies. CNV and gene expression analysis revealed a significant variability between NCI-H1581 and NCI-H1703, which could suggest differences in the molecular mechanism underlying the emergence of acquired resistance to FGFR inhibitor. Subsequently, the resistant variants of these cells were developed in order to investigate a possible molecular mechanism of acquired resistance to FGFR inhibitor. We found that both CPL304110-resistant cell lines (NCI-H1581R and NCI-H1703R) were characterised by “switched off” FGFR signalling. Although several studies indicated the reactivation of PI3K/AKT and MAPK pathways as a possible bypass mechanism of resistance to FGFR inhibition [15,27,28,35], we did not observe any significant changes in the expression/phosphorylation level of AKT or ERK1/2 in resistant cell lines. Interestingly, both NCI-H1581R and NCI-H1703R demonstrated an increase in expression and phosphorylation of p38 kinase. Moreover, the ectopic overexpression of p38 kinase in CPL304110-sensitive cells conferred the resistance to the inhibitor, whereas the inhibition of p38 activity with SB202190 and SB203580 led to CPL304110-mediated growth suppression of both resistant variants. Overall, p38 kinase is activated in response to a variety of extracellular stimuli and is mainly described as a stress-activated kinase. In accordance with our observations, a previous study showed that dual inhibition of EGFR and p38 had a synergistic inhibitory effect on the proliferation of bladder cancer cell lines [36]. Moreover, another research unveiled that p38 MAPK confers intrinsic resistance to EGFR TKIs, lapatinib and gefitinib, in K-Ras mutant colon cancer cell lines, by concurrent stimulation of EGFR gene transcription and protein dephosphorylation [37]. In NSCLC cell lines and a mouse PDX model, Yeung et al. showed that acquired resistance to gefitinib is mediated by YAP-MKK3/6-p38 MAPK-STAT3 signalling, and both inhibition and knockdown of p38 result in cell resensitisation and overcoming resistance [38]. Meanwhile, Malchers et al. reported that PD173074, a pan-FGFR inhibitor, induced apoptosis in lung cancer cells overexpressing both FGFR1 and MYC but not FGFR1 alone, suggesting that MYC is required for cell response to FGFR inhibition [39]. These results are in concordance with our data demonstrating a decrease in CNV and gene expression of both *FGFR1* and *MYC* in resistant variants of NCI-H1581 and NCI-H1703. It was also reported that the mechanism of resistance to FGFR inhibitor was accompanied by *NRAS* amplification and *DUSP6* deletion (member of a subfamily of protein tyrosine phosphatases known as dual-specificity phosphatases, negative regulators of MAPK signalling [40]), which led to the MAPK pathway reactivation [14]. Moreover, the silencing of *DUSP6* led to an increase in p38 phosphorylation [41]. Interestingly DUSP6-mediated negative regulation of p38 has been demonstrated in hepatocellular carcinoma, where enhanced polyubiquitination and the degradation of DUSP6 contributed to the increased phosphorylation of p38. This led to cell proliferation and cell cycle progression of cancer cells via activation of p38 pathway [42]. In line with these studies, we observed that both resistant cell variants showed a significant decrease in *DUSP6* gene expression with a subsequent increase in p38 protein expression. These results confirmed that the bypass mechanism of resistance to CPL304110 in these cell lines is driven by p38 kinase. We can speculate that resistance to FGFR inhibition is mediated by MAPK pathway activation, possibly in concert with the loss of DUSP6. Taking into account that our RNA-sequencing analyses of p38 signalling revealed a panel of 54 genes common for both analysed cell lines with the same pattern of changes in expression following the acquisition of resistance to CPL304110, there are several other gene candidates possibly involved in the mechanism of FGFR inhibition resistance. Among these genes, members of MAPK, TGFβ, PI3K, EGF, and FGF pathways, as well as genes involved in the regulation of cell cycle and apoptosis, were found. Further evaluation of these pathways would be required to identify the complexity of the resistance mechanism.

Herein, for the first time, we demonstrate that despite initial genomic and transcriptomic differences between CPL304110-sensitive cell lines revealed by CNV analysis by aCGH and RNA sequencing, in both NCI-H1581 and NCI-H1703 cells, activation of p38 kinase is involved in the development of acquired resistance to FGFR inhibition. In conclusion, targeting p38 activity may be a potential therapeutic approach to circumvent FGFR inhibitor resistance.

## Data Availability

The data presented in this study are available in the article and Supplementary Materials. Further inquiries can be directed to the corresponding authors. Sequencing data have been deposited in GEO under accession codes: GSE189270 (https://www.ncbi.nlm.nih.gov/geo/query/acc.cgi?acc=GSE189270, accessed on 23/11/2021); GSE189278 (https://www.ncbi.nlm.nih.gov/geo/query/acc.cgi?acc=GSE189268, accessed on 23/11/2021); GSE189269 (https://www.ncbi.nlm.nih.gov/geo/query/acc.cgi?acc=GSE189269, accessed on 23/11/2021).

## Supplementary Materials

The following are available online at https://www.mdpi.com/article/10.3390/cells10123363/s1, Figure S1: Quantitative analysis of NCI-H1581 cells grown in 3D BD Matrigel® in the presence of CPL304110; Figure S2: Quantitative analysis of NCI-H1581 and cells grown in 3D BD Matrigel®; Western Blot analysis of proteins involved in cell cycle regulation; Analysis of variance between sensitive and CPL304110-resistant cells; Figure S3: Western blot analysis of p38 protein expression in all tested cell lines; Figure S4: Hierarchical clustering of gene sets/pathways related to p38, p38 MAPK pathway, MAPK pathway, and p38 substrates; Figure S5: Western blot analysis was performed to assess phosphorylation of p38 in sensitive and resistant cell lines upon SB202190 treatment; Figure S6: Quantitative analysis of NCI-H1581 and NCI-H1581R cells lines grown in 3D BD Matrigel® in the presence of CPL304110 and/or SB202190; Figure S7: Colony formation assay was performed for sensitive and resistant cells treated with CPL304110 and/or SB202190; Figure S8: Sensitive and resistant variants of NCI-H1581 and NCI-H1703 cells were grown with CPL304110 and/or SB203580 in 3D BD Matrigel®; Figure S9: Quantitative analysis of NCI-H1581, NCI-H1581R, and NCI-H1581/p38↑ cells grown in 3D BD Matrigel® in the presence of CPL304110.
