# Supplementary material for: p38 Mediates Resistance to FGFR Inhibition in Non-Small Cell Lung Cancer
Source: Cells. 2021 Nov 30;10(12):3363. doi: 10.3390/cells10123363 (PMC8699485; doi:10.3390/cells10123363)
Supplement: Supplementary file 1 [file cells-10-03363-s001.zip › Supplementary Figures description REVISION.pdf]

Figure S1: Quantitative analysis of NCI-H1581 cells grown in 3D BD Matrigel® in the presence of CPL304110 in indicated concentrations. Colonies were measured and statistically analysed with ImageJ. Data are expressed as mean ± SD, *** $p \leq 0.001$, $n = 3$.

Figure S2: (A) Quantitative analysis of NCI-H1581 and NCI-H1581R cells grown in 3D BD Matrigel®, treated with CPL304110 in indicated concentrations. Colonies were measured and statistically analysed with ImageJ. Data are represented as mean ± SD, ***$p \leq 0.001$, $n = 3$. **(B)** Western blot analysis was performed to assess the changes in expression and phosphorylation of proteins involved in cell cycle regulation in sensitive and resistant cell lines upon CPL304110 treatment (0.1 μM for NCI-1581 and NCI-H1581R; 1 μM for NCI-1703 and NCI-H1703R) for 48 h. Experiments were conducted in triplicates. Representative blots are shown. **(C)** Principal Component Analysis (PCA) was performed on all samples (NCI-H1581 and NCI-H1703 cell lines in the sensitive (S) and resistant (R) variants). PC1-PC4 explain 95% of the variability in gene expression profile within the analyzed set of samples. None of these PCs clearly distinguishes cell variants (S versus R). Instead, samples tend to group by the cell line. This difference is most prominent (74% of variance) and is described by the first principal component (PC1). **(D)** A heat map representing hierarchical clustering for the top 500 differentially expressed genes (genes with the highest variances in the analyzed dataset; data subjected to rlog transformation). Heat map demonstrates that these genes have a distinct expression in NCI-H1581 versus NCI-H1703 cells, but ubiquitous expression irrespectively from cell line variant (sensitive versus resistant).

Figure S3: Western blot analysis of p38 protein expression was performed for lysates of all five lung cancer cell lines. Experiments were conducted in triplicates. Representative blots are shown.

Figure S4: (A-F) Hierarchical clustering of gene sets/pathways related to p38: (**A**: NCI-H1581, **B**: NCI-H1703) p38 MAPK pathway (**C**: NCI-H1581, **D**: NCI-H1703) MAPK pathway, (**E**: NCI-H1581, **F**: NCI-H1703) List of p38 substrates according to Trempolec et al., 2013 (43).

Figure S5: Western blot analysis was performed to assess phosphorylation of p38 in sensitive and resistant cell lines upon SB202190 (2 μM) treatment for 48 h. Experiments were conducted in triplicates. Representative blots are shown.

Figure S6: Quantitative analysis of NCI-H1581 and NCI-H1581R cells lines grown in 3D BD Matrigel® in the presence of CPL304110 (0.1 μM) and/or SB202190 (2 μM). Colonies were

measured and statistically analysed with ImageJ. Data are represented as mean ± SD, ***$p \leq 0.001$, n = 3.

Figure S7: Colony formation assay was performed for sensitive and resistant cells treated with CPL304110 (0.1 µM for NCI-H1581 and NCI-H1581R; 1 µM for NCI-H1703 and NCI-H1703R and/or SB202190 (2 µM). Cells were cultured for 21 days, fixed colonies were stained with 0.4% crystal violet, and photographed using Optilia W30x-HD camera for visual assessment. Experiments were conducted in triplicates. Representative pictures are shown.

Figure S8: Sensitive and resistant variants of NCI-H1581 and NCI-H1703 cells were grown with CPL304110 (0.1 µM for NCI-H1581 and NCI-H1581R; 1 µM for NCI-H1703 and NCI-H1703R) and/or SB202190 (2 µM) in 3D BD Matrigel®. Cell growth was measured with ImageJ software after 14 days of culture. Representative pictures were taken. Scale bar represents 100 µm, n = 3.

Figure S9: Quantitative analysis of NCI-H1581, NCI-H1581R, and NCI-H1581/p38↑ cells grown in 3D BD Matrigel® in the presence of CPL304110 (0.1 µM). Colonies were measured and statistically analysed with ImageJ. Data are represented as mean ± SD, ***$p \leq 0.001$, n = 3.
