# Supplementary figures and images for: p38 Mediates Resistance to FGFR Inhibition in Non-Small Cell Lung Cancer
Source: Cells. 2021 Nov 30;10(12):3363. doi: 10.3390/cells10123363 (PMC8699485; doi:10.3390/cells10123363)

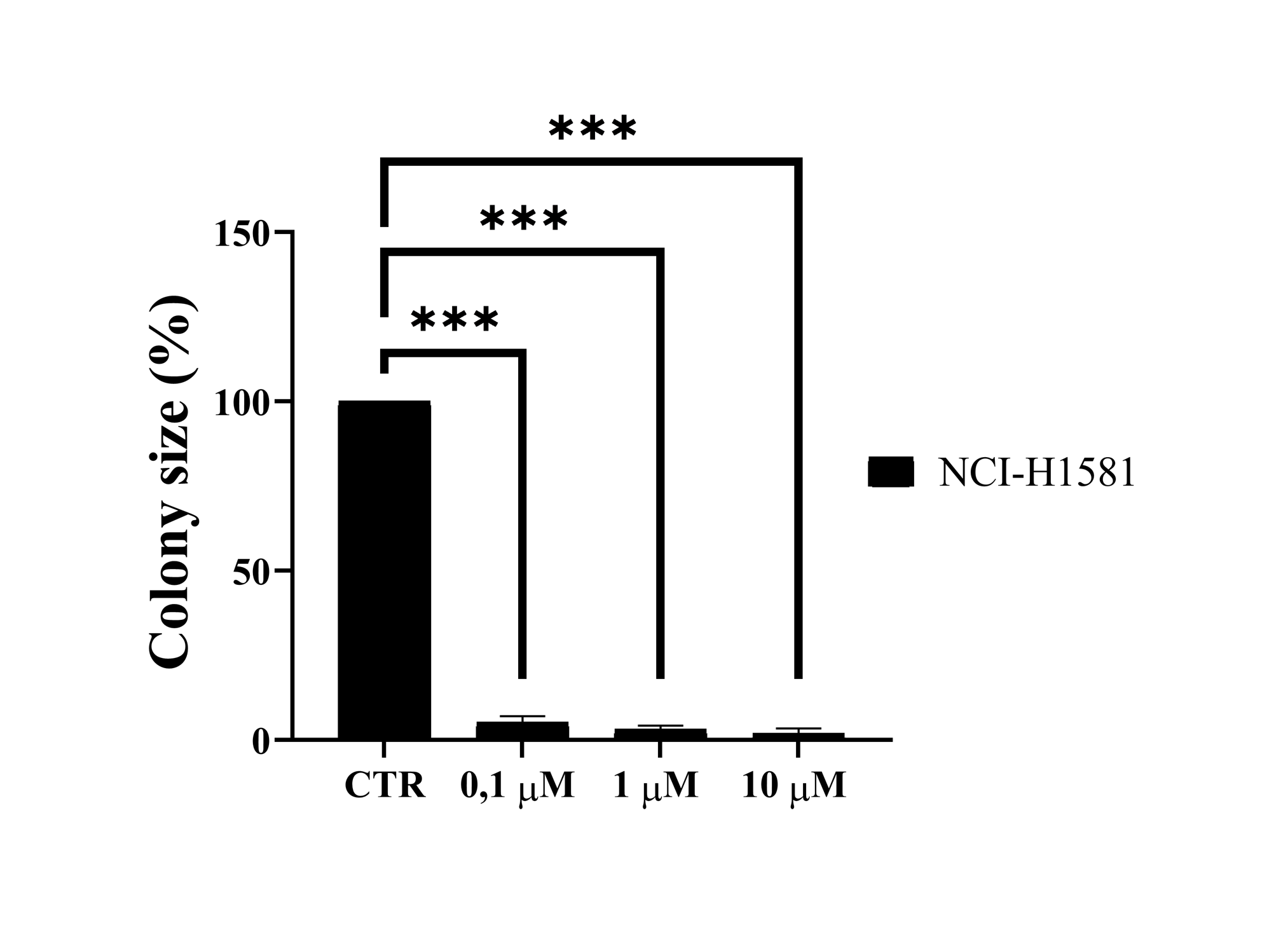

Supplement: Supplementary file 1 [file cells-10-03363-s001.zip › Supplementary Figure S1.tif]

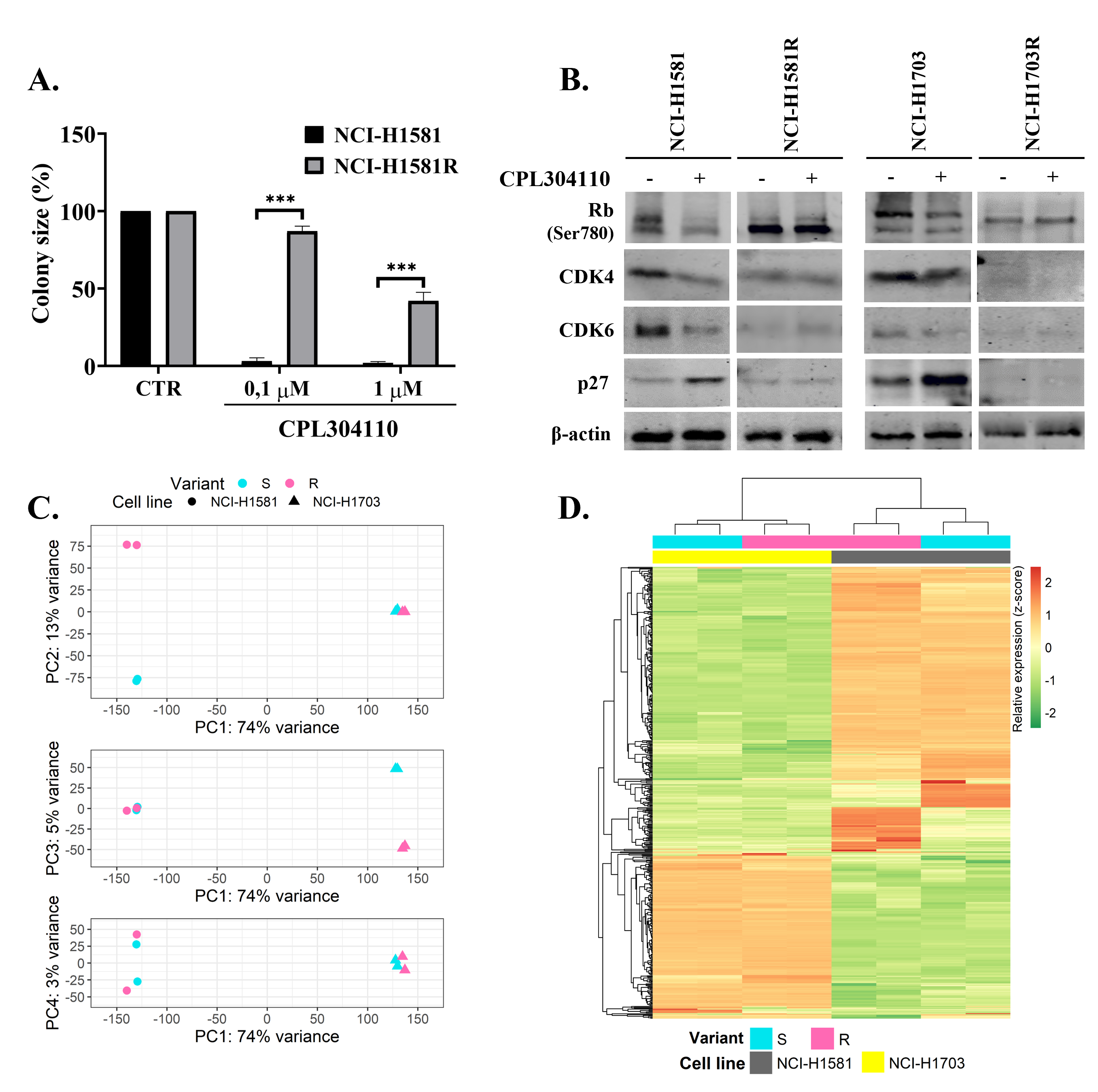

Supplement: Supplementary file 1 [file cells-10-03363-s001.zip › Supplementary Figure S2.tif]

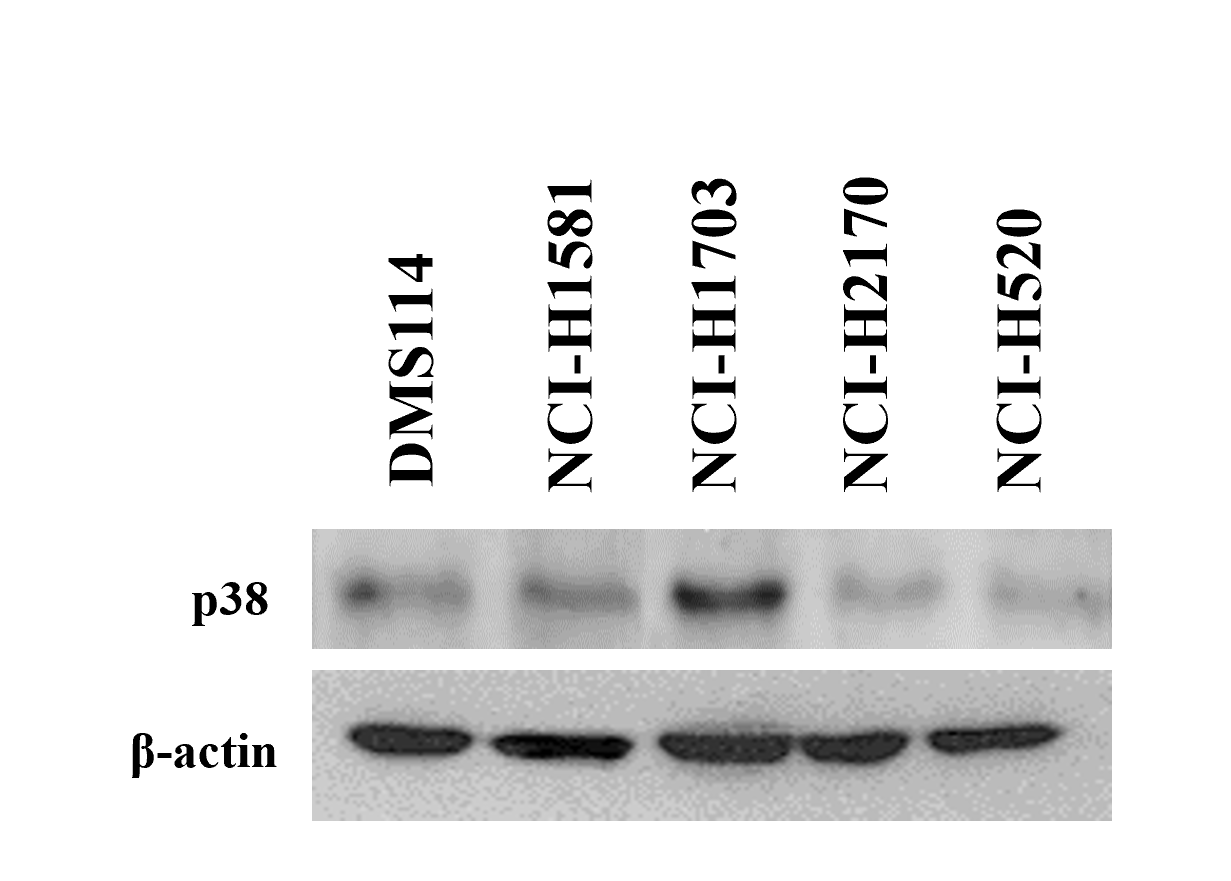

Supplement: Supplementary file 1 [file cells-10-03363-s001.zip › Supplementary Figure S3.tif]

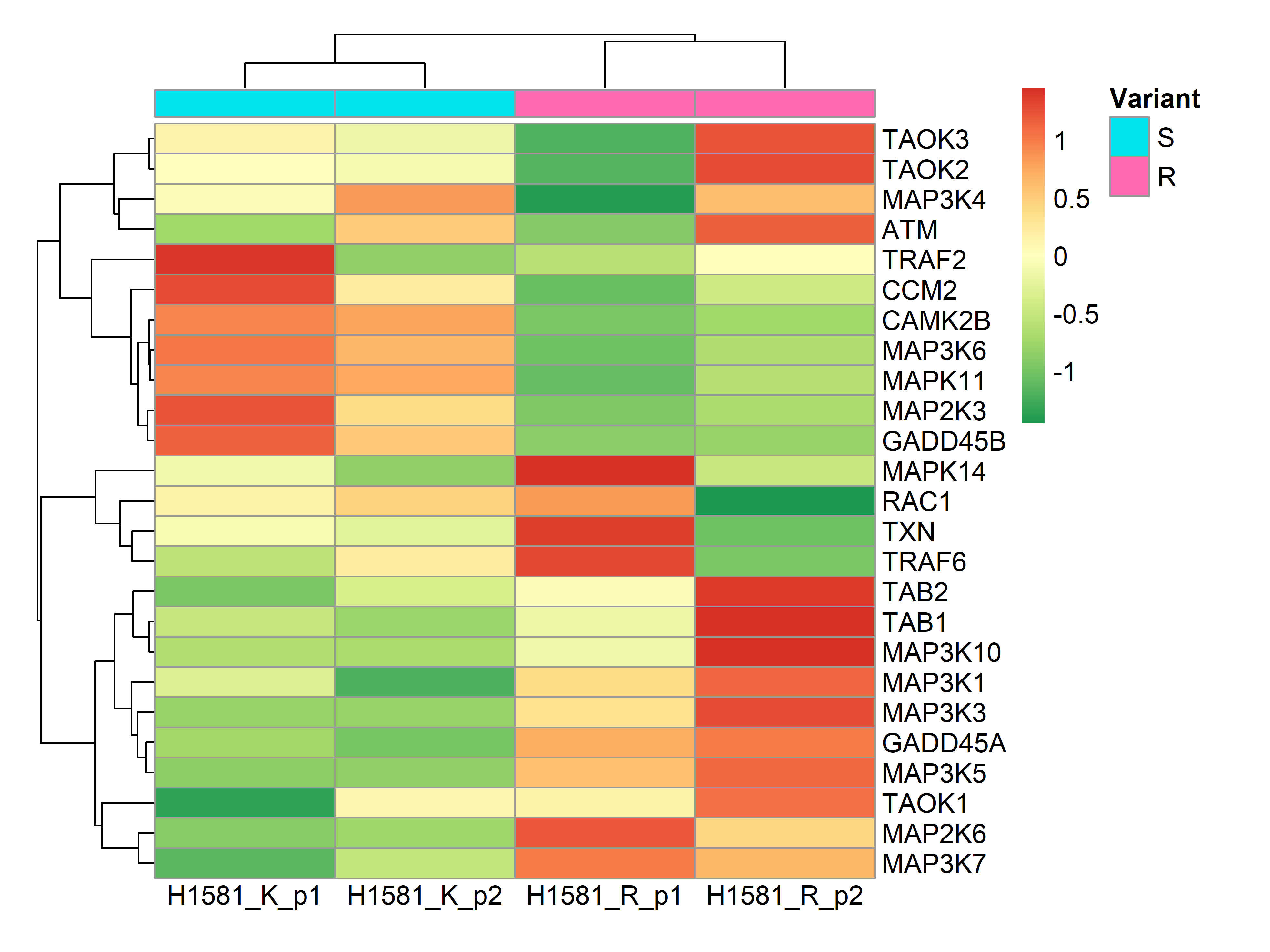

Supplement: Supplementary file 1 [file cells-10-03363-s001.zip › Supplementary Figure S4A.tiff]

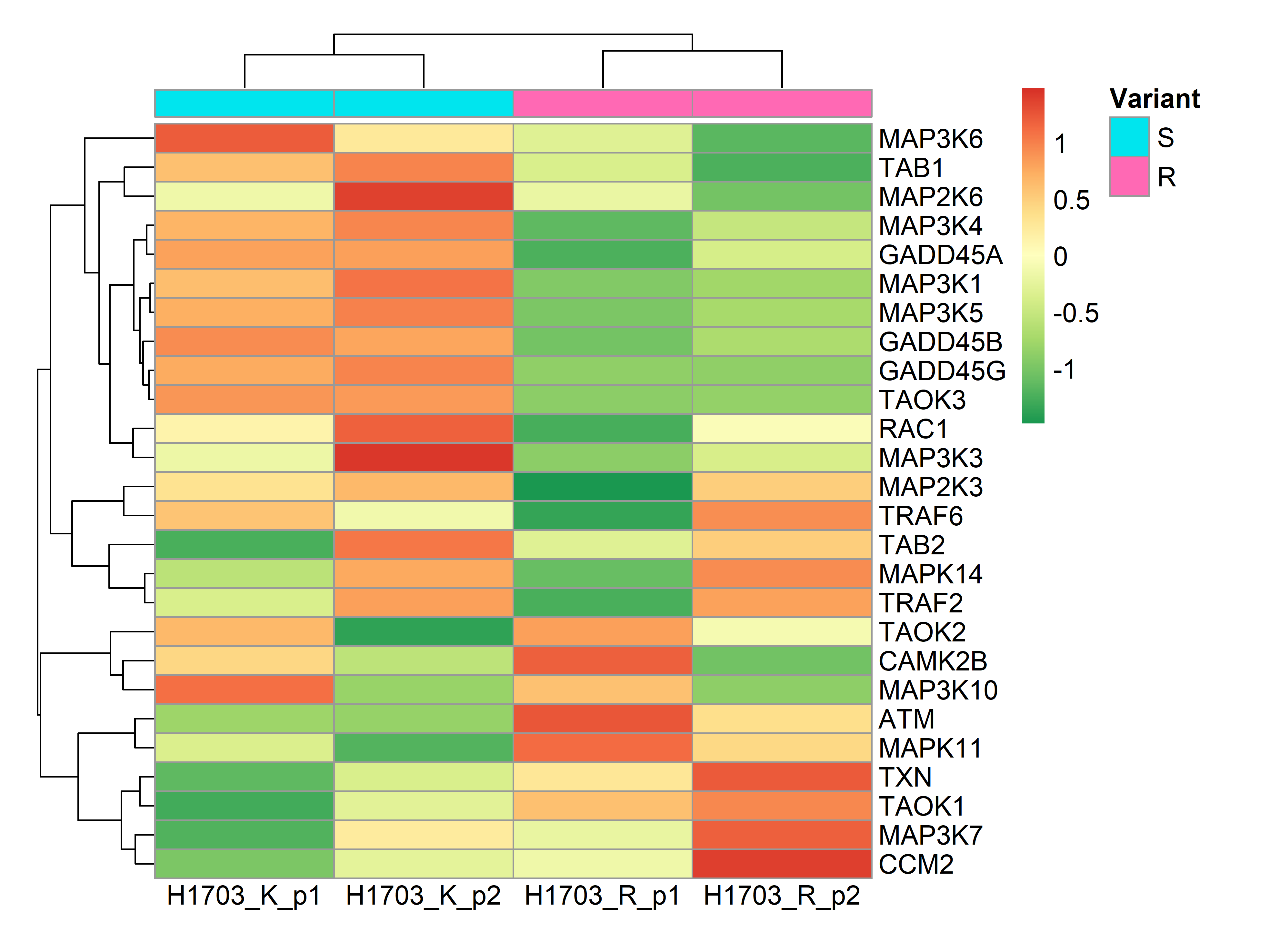

Supplement: Supplementary file 1 [file cells-10-03363-s001.zip › Supplementary Figure S4B.tiff]

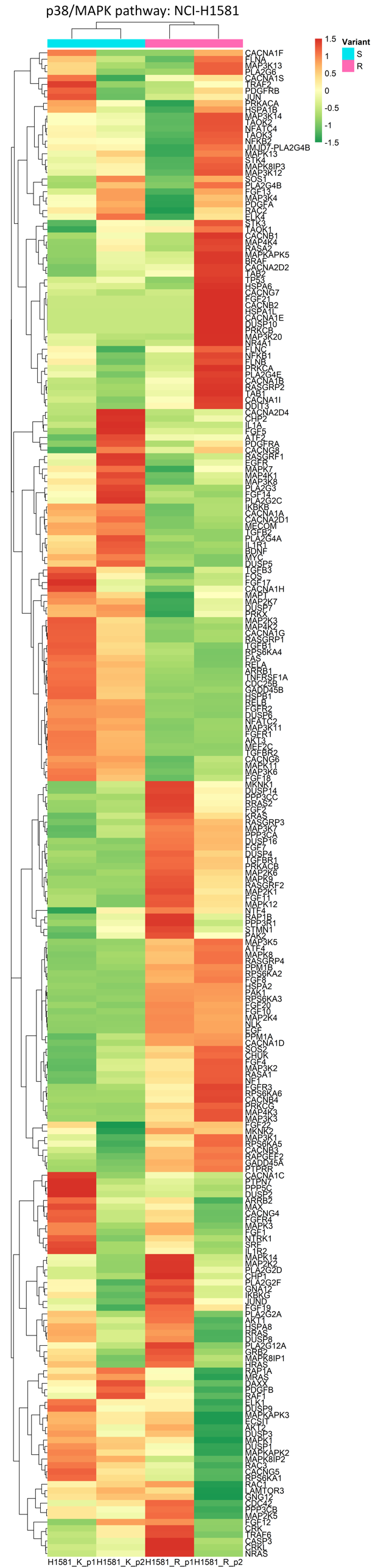

Supplement: Supplementary file 1 [file cells-10-03363-s001.zip › Supplementary Figure S4C.tiff]

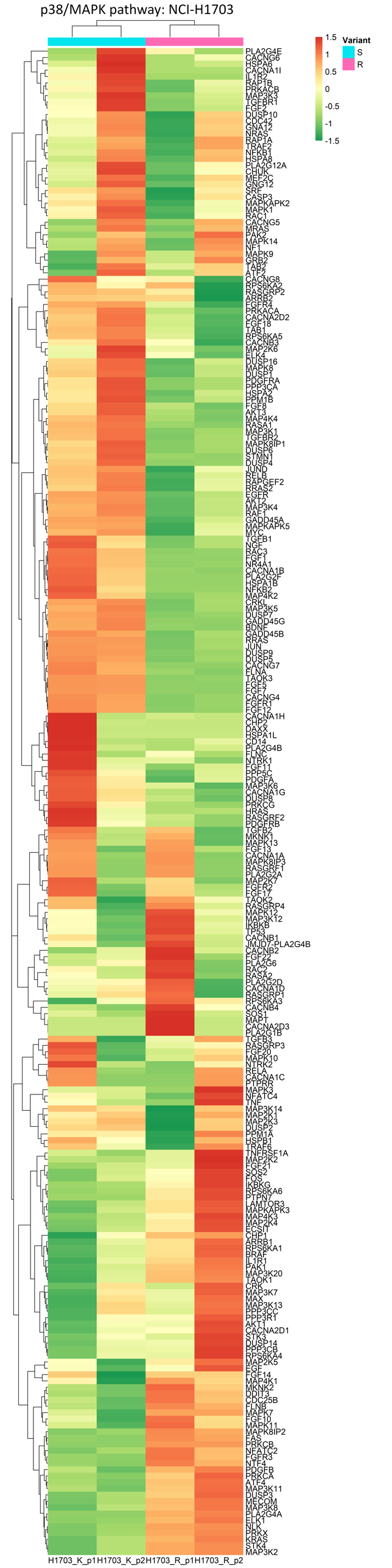

Supplement: Supplementary file 1 [file cells-10-03363-s001.zip › Supplementary Figure S4D.tiff]

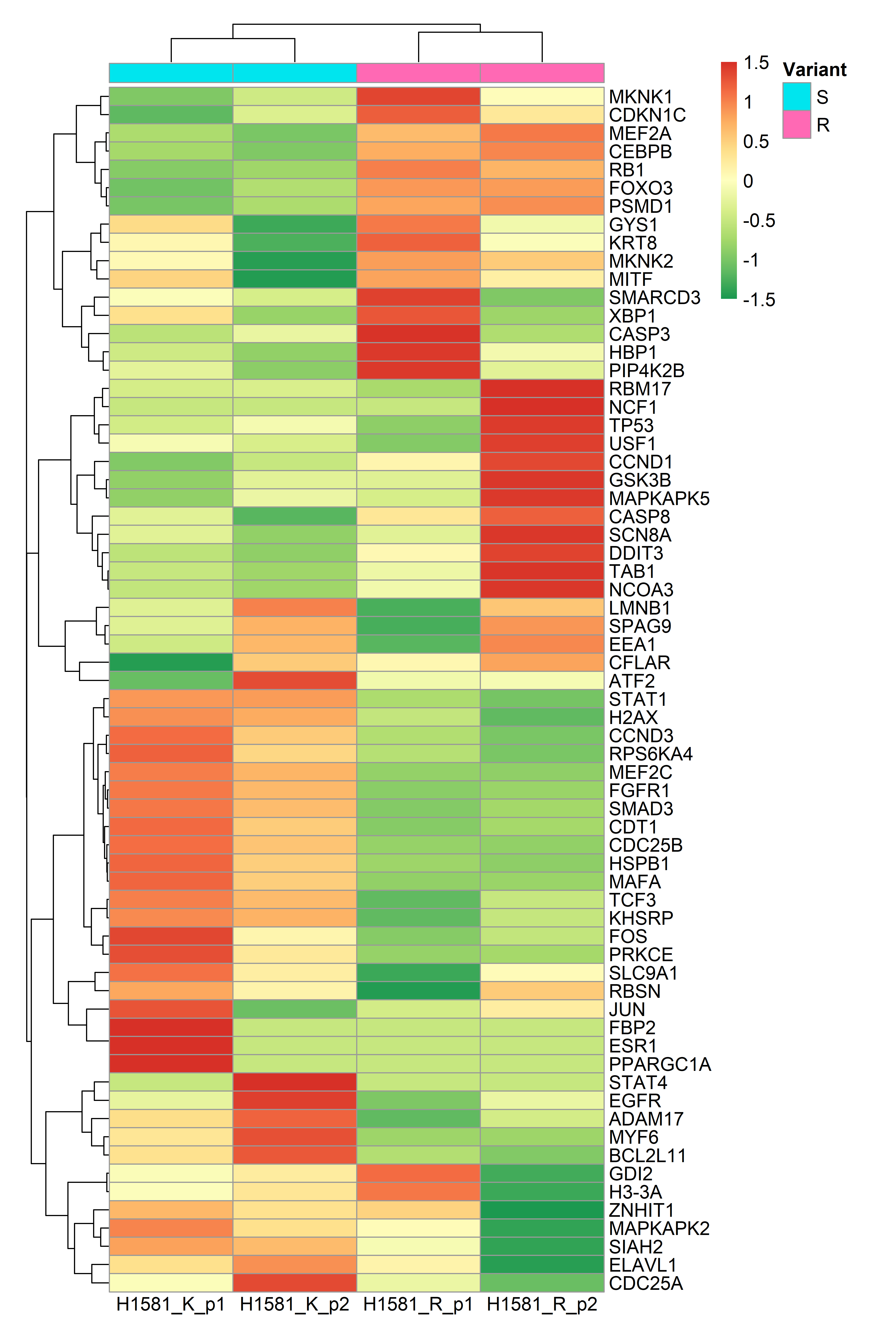

Supplement: Supplementary file 1 [file cells-10-03363-s001.zip › Supplementary Figure S4E.tiff]

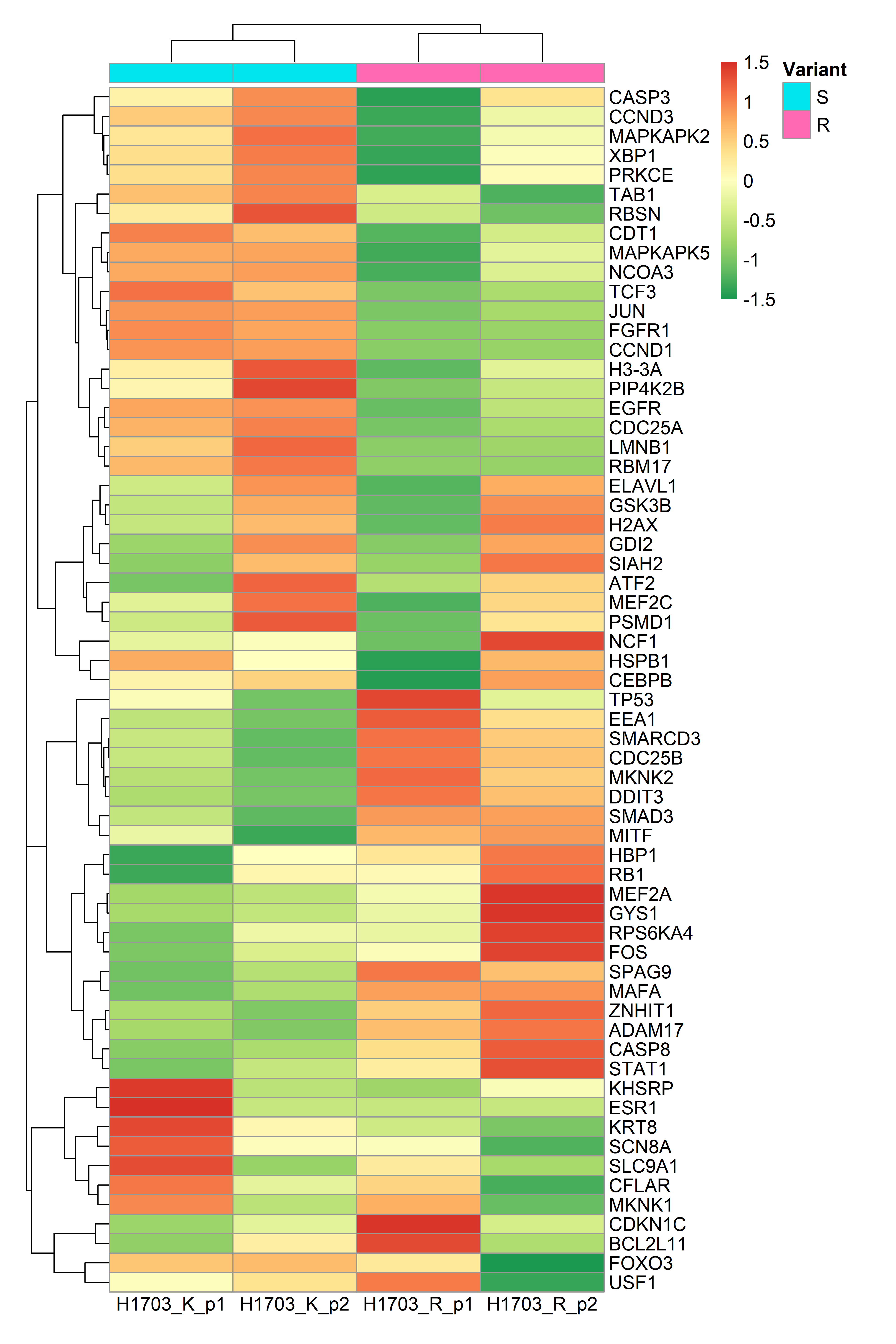

Supplement: Supplementary file 1 [file cells-10-03363-s001.zip › Supplementary Figure S4F.tiff]

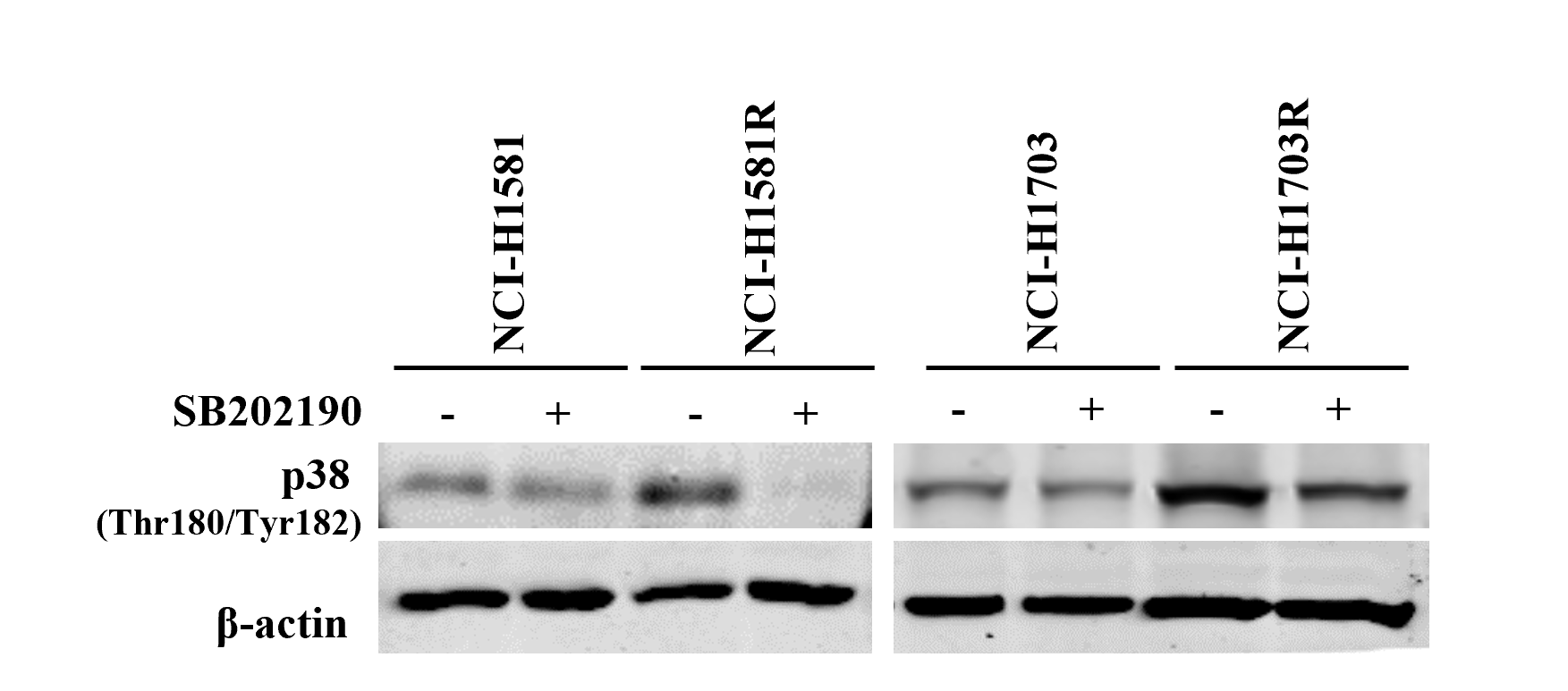

Supplement: Supplementary file 1 [file cells-10-03363-s001.zip › Supplementary Figure S5.tif]

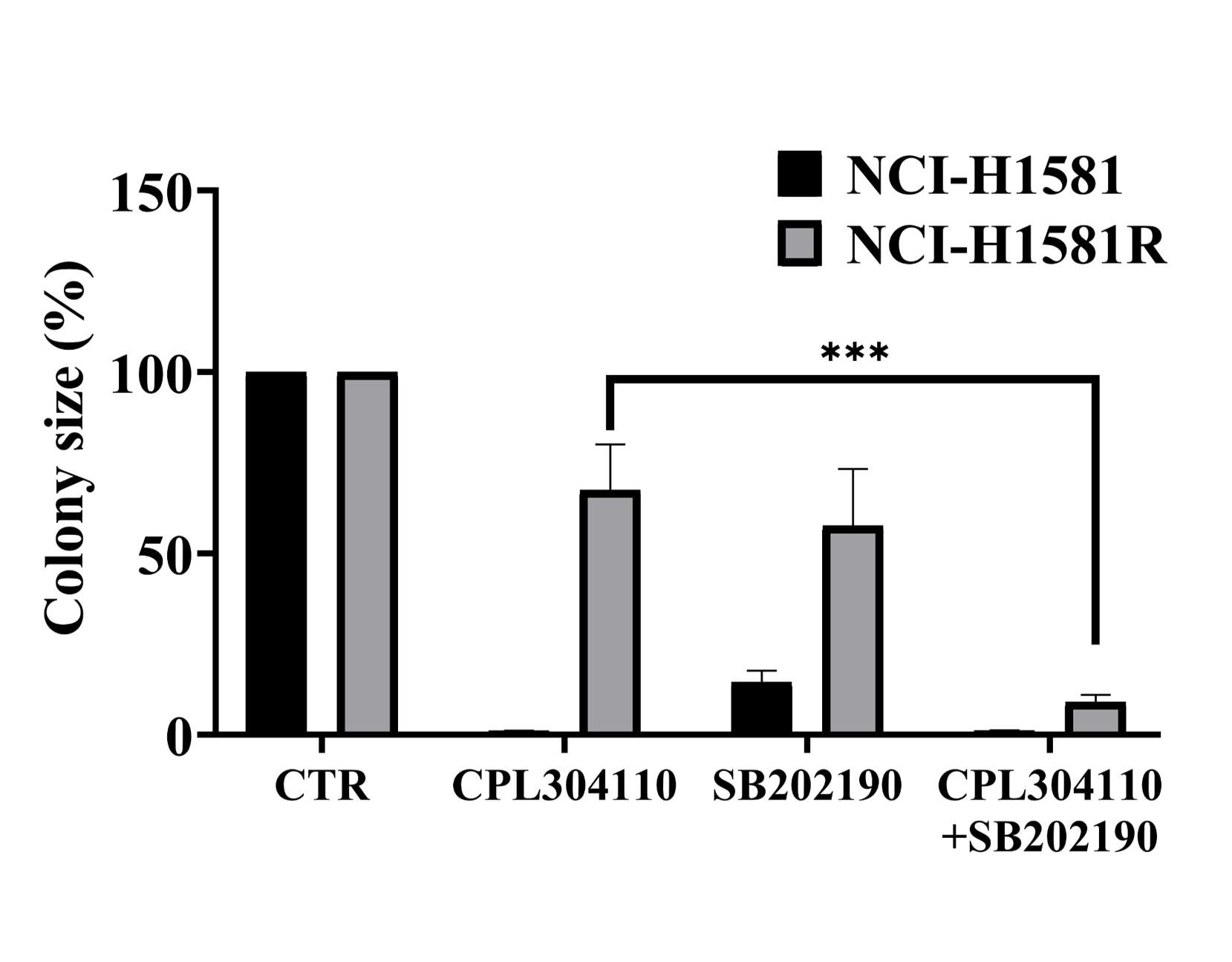

Supplement: Supplementary file 1 [file cells-10-03363-s001.zip › Supplementary Figure S6.tif]

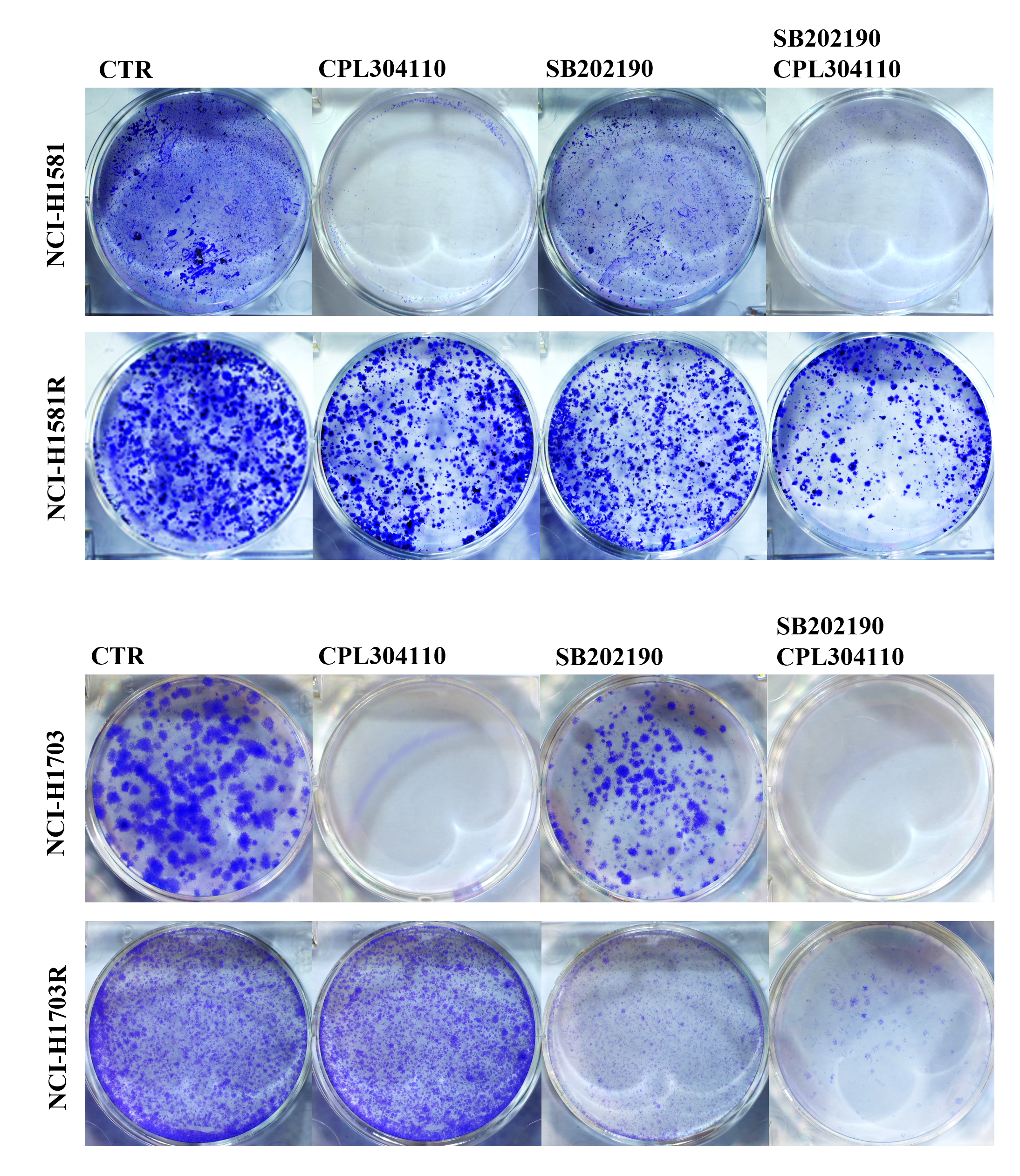

Supplement: Supplementary file 1 [file cells-10-03363-s001.zip › Supplementary Figure S7.tif]

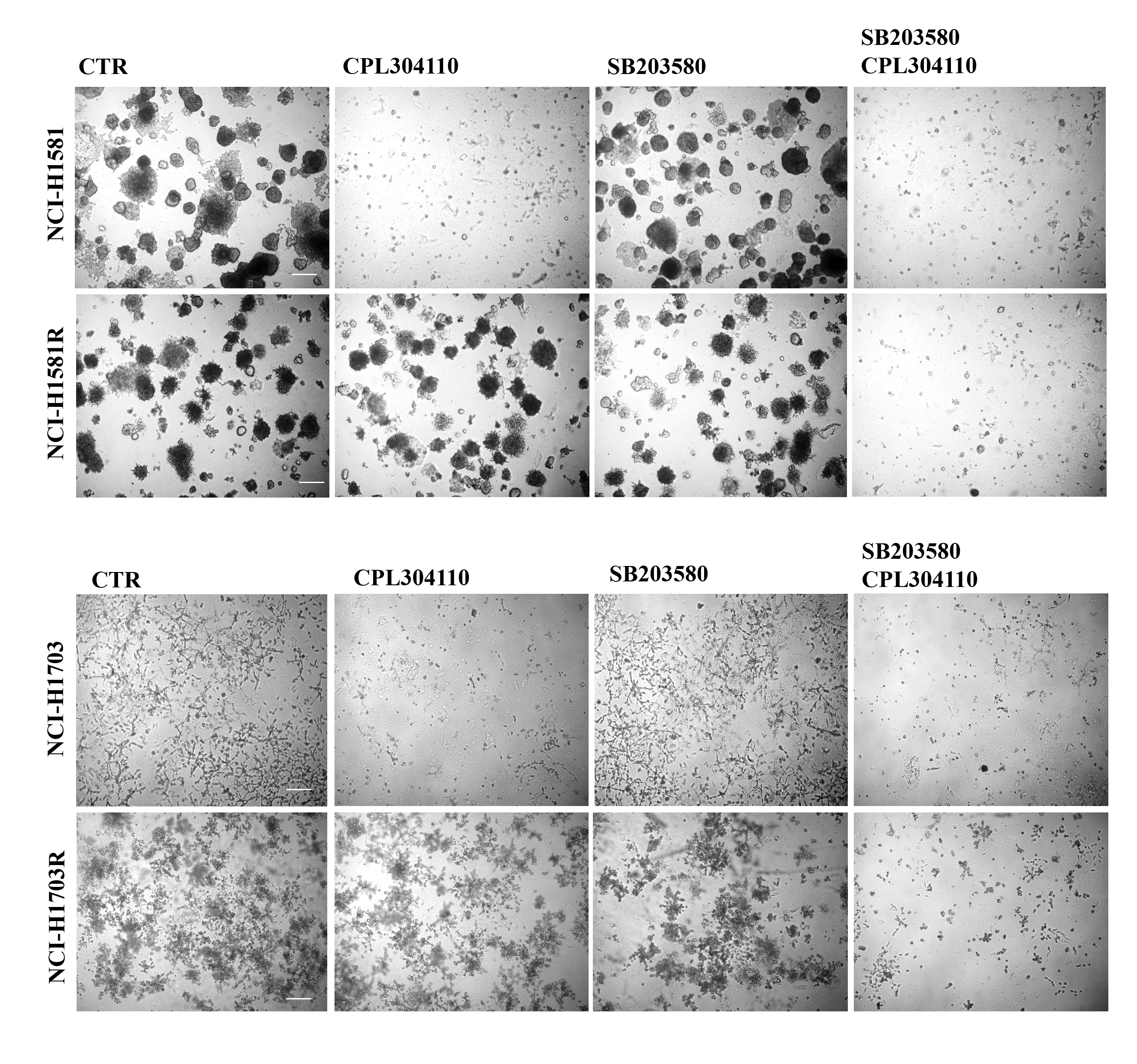

Supplement: Supplementary file 1 [file cells-10-03363-s001.zip › Supplementary Figure S8.tif]

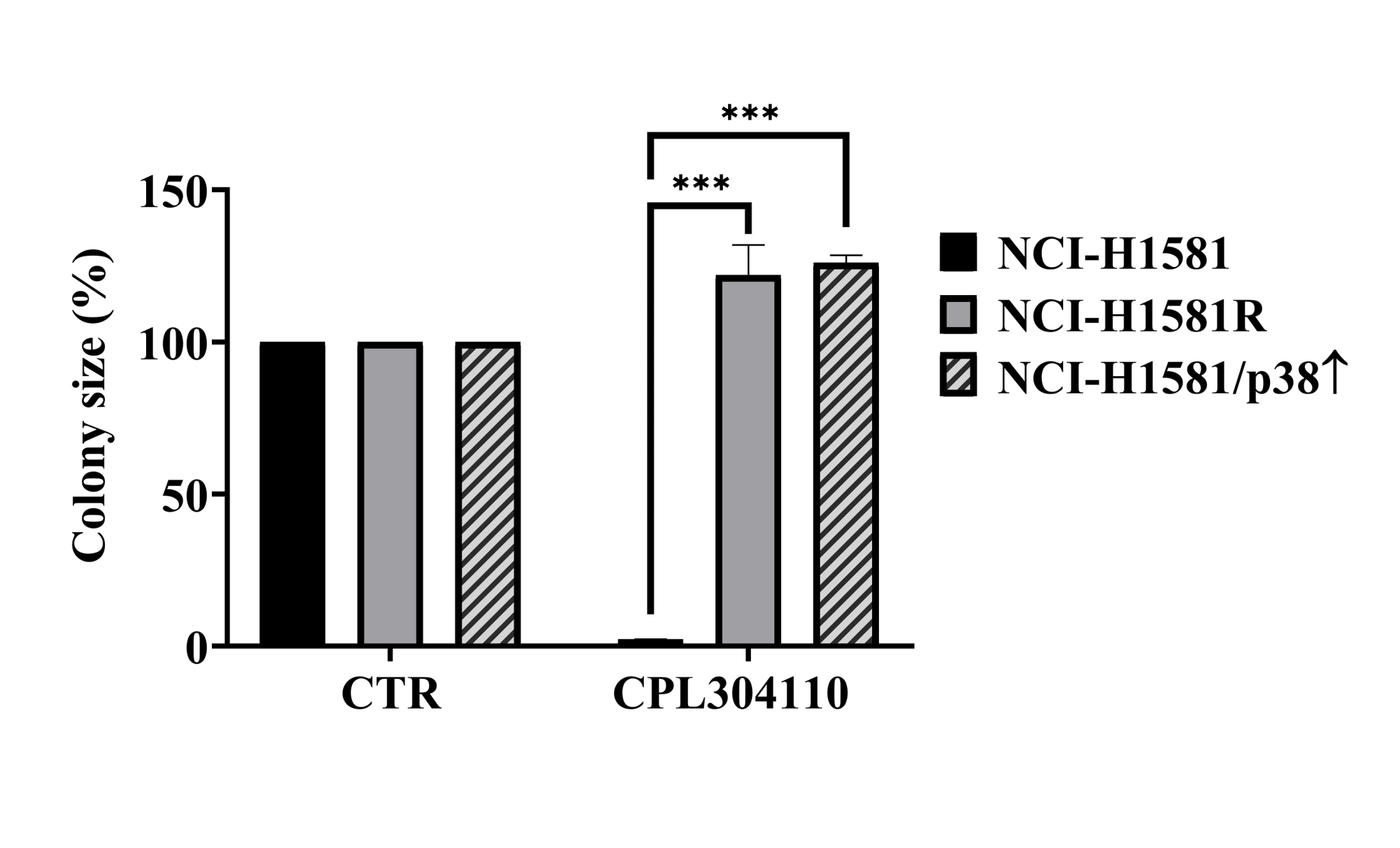

Supplement: Supplementary file 1 [file cells-10-03363-s001.zip › Supplementary Figure S9.tif]

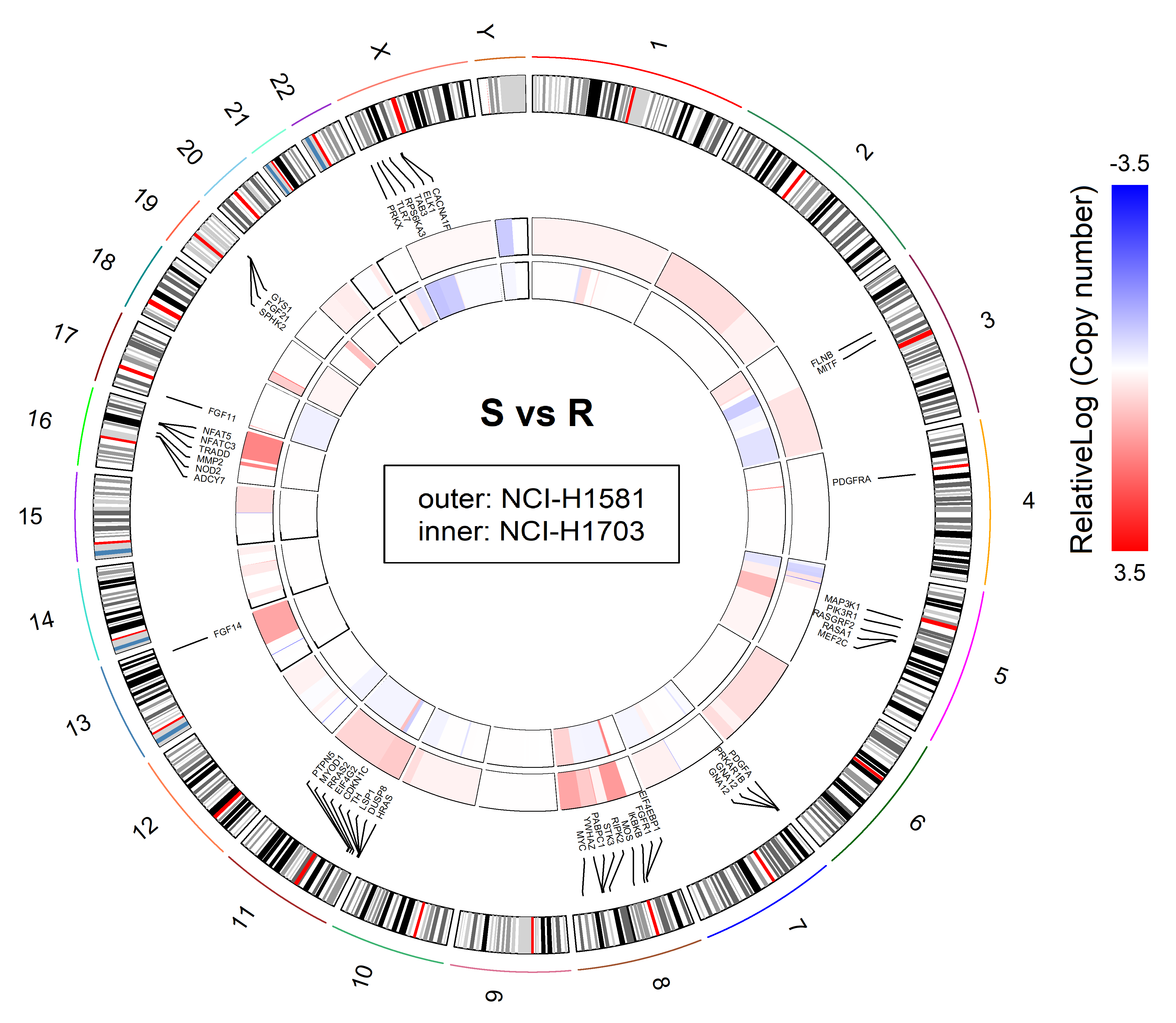

Supplement: Supplementary file 1 [file cells-10-03363-s001.zip › Supplementary Materials Figure 3B - high resolution.tif]
